# Biological characteristics of γδT cells and application in tumor immunotherapy

**DOI:** 10.3389/fgene.2022.1077419

**Published:** 2023-01-04

**Authors:** Renhong Zhu, Qian Yan, Yashu Wang, Keqiang Wang

**Affiliations:** ^1^ Department of Laboratory Medicine, Second Affiliated Hospital of Shandong First Medical University, Tai’an, China; ^2^ Department of Laboratory Medicine, Tai’an Tumor Prevention and Treatment Hospital, Tai’an, China; ^3^ Department of Laboratory Medicine, Second Hospital of Traditional Chinese Medicine, Tai’an, China; ^4^ Department of Laboratory Medicine, The Affiliated Tai’an City Central Hospital of Qingdao University, Tai’an, China

**Keywords:** γδT cells, tumor, immunotherapy, progress, biological

## Abstract

Human γδT cells are a special immune cell type which exist in small quantities in the body, do not require processing and presentation for antigen recognition, and have non-major histocompatibility complex (MHC)-restricted immune response. They play an important role in the body’s anti-tumor, anti-infection, immune regulation, immune surveillance and maintenance of immune tolerance. This article reviews the generation and development of human γδT cells, genetic characteristics, classification, recognition and role of antigens, and research progress in tumor immunotherapy.

## 1 Introduction

T cells are classified into αβT cells and γδT cells according to the differences in the types of their cell receptors (T cell receptor, TCR). γδT cells are considered to be special immune cells between acquired immunity and natural immunity due to their distribution characteristics in the body and the non-MHC restricted characteristics of immune response. They both play a unique role in innate immunity and in acquired immunity. The role of the response is gradually revealed. Numerous studies have shown that γδT cells play an important role in the body’s anti-infection, anti-tumor, immune surveillance and regulation (Wang K. Q et al., 2017; Wang et al., 2018; Wang Y. R et al., 2019; Wang Y. S et al., 2019; Zhao et al., 2021a; Zhao et al., 2021b). This article reviews the generation and development of human γδT cells, genetic characteristics, classification, recognition and role of antigens, and research progress in tumor immunotherapy.

## 2 Biological characteristics of γδT cells

### 2.1 The production and development of γδT cells

Human γδT cells occur in the thymus medulla of normal fetuses at 7–8 weeks, and their development process are similar to that of αβT cells. Before gaining autoimmune tolerance, they not only need to undergo functional TCR expression, but also need to undergo negative selection. The point is that some γδT cells have not undergone double positive selection, so that γδT cells have non-limiting MHC when recognizing antigens. Studies have found that the formation of various functions of γδT cells begins in the thymus and matures in the peripheral blood. In the thymus, thymic precursor cells differentiate into γδTCR + thymocytes under the control of the TCR signal pathway. After leaving the thymus, they enter the peripheral blood circulation and become γδT cells in the peripheral blood circulation. So far, the immune function of various γδT cell subtypes has been basically perfected. Subsequently, these cells can differentiate into a single oligoclonal cell subtype under the induction of TCR ligand-related molecules, and further develop under the action of various hormones released by the thymus, and finally have the function of mature immune cells (Cook et al., 2008; Narayan et al., 2012; Shekhar et al., 2012).

### 2.2 The genetic characteristics of γδT cells

The TCRγδ gene contains four groups of genes V (variable region), D (diversity region), J (joining region) and C (constant region). The *γ* chain gene consists of 10 V gene segments, 2 D gene segments, Two J gene segments are composed of C, while the delta chain gene is composed of only 7 V gene segments, 2 J gene segments and C. The combination of these gene segments and the diversity of junction regions make TCRγδ have the potential for diversity. However, because γδT cell subsets often only use a specific combination of VγVδ and J region sequences, the TCRγδ structure lacks diversity (Chen et al., 2012). The gene recombination and matching of γδT cells are highly coordinated. Vγ9 and Cγ1 are linked and almost all combine with Vδ2 to form TCR dimers. Studies have confirmed that the Vγ9Vδ2 subtype in adult peripheral blood accounts for more than 90% of the total number of γδT cells (Yang et al., 2011). Therefore, the γδT gene lacks diversity with limited gene rearrangement, MHC non-restrictive reaction with antigen (Davey et al., 2018), and similar functions to cells related to natural immunity, so it was initially considered to be an important part of the body’s innate immunity.

### 2.3 Recognition and effect of γδT cells on antigen

**FIGURE 1:**
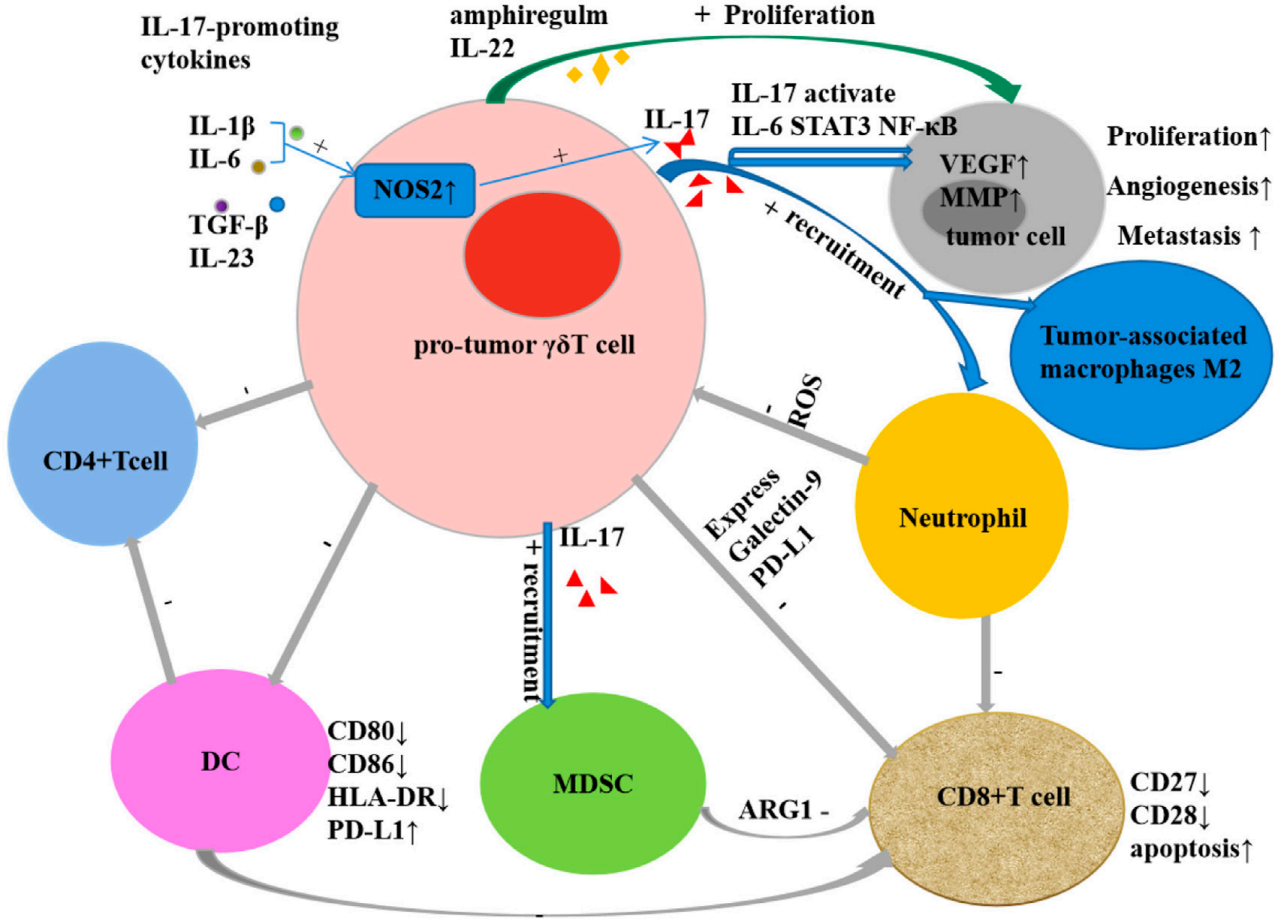
The tumor-promoting effect of γδT cells.

**FIGURE 2:**
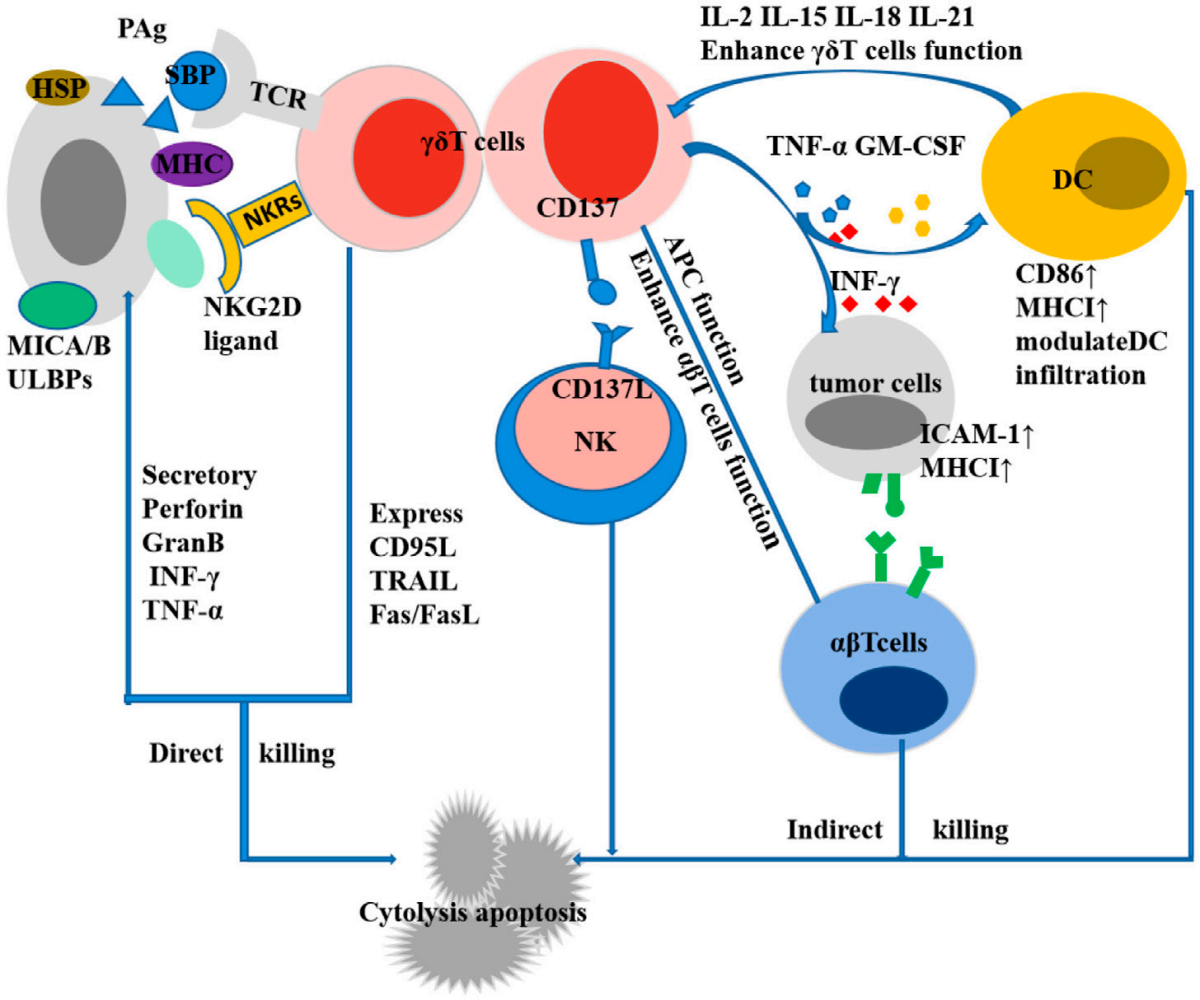
Anti-tumor effect of γδT cells.

γδT cells are not only an important cell group involved in innate immune response, but also a key component of non-specific immune response. The recognition of γδT cells to antigens is not restricted by MHC and can directly recognize antigens. Not only can they recognize complete polypeptides, they can respond to certain MHC-like molecules, and they also show special affinity for heat shock proteins. The recognition of γδT cells to antigens shows certain tissue specificity: γδT cells from the same tissue express the same TCR to recognize antigens of the same nature, while γδT cells from different tissues can express different TCRs to recognize antigens of different properties (Khatri et al., 2010). The antigens recognized by γδT cells currently found mainly include MHC and MHC-like molecules, heat shock proteins (HSP), DNA mismatch repair related proteins (MSH2), phosphorylated antigens, and those presented by CD1a, CD1c, and CD1d in the CD1 family Lipid antigens and so on (Sebestyen et al., 2020). These antigens bind to T cell receptors or NK cell receptors on the surface of γδT cells to cause the activation of γδT. Natural killer receptors (NKR) and Toll-like receptors (TLR) can provide costimulatory signals to participate in the activation process. Parts of the mechanisms of the activation of Vγ9Vδ2+ T cells by phosphoantigens are mediated through the B7 immunoglobulin family-like butyrophilin 2A1 (BTN2A1) and BTN3A1 complexes. Following phosphoantigen binding to the intracellular B30.2 domains of BTN3A1 in tumor cells, BTN3A1 undergoes a conformational change (Sandstrom et al., 2014; Gu et al., 2017) and promotes the interaction between BTN2A1 and BTN3A1 intracellular domains (Rigau et al., 2020). Subsequently, the germline-encoded regions of the TCR Vγ9 chain directly bind to BTN2A1 on tumor cells (Rigau et al., 2020), then leads to Vγ9Vδ2+ T Cell activation. Activated γδT cells exhibit a variety of biological and immunological functions: 1) Non-specific immune response: Without the presentation of APC, it can be activated directly through TCR to recognize multiple antigen components, participate in non-specific immune response and play an important role. 2) Secretion of a variety of cytokines: by secreting cytokines such as TNF-α, IFN-γ, IL-4, IL-10, etc., it can not only directly inhibit tumor growth, but also promote the maturation of dendritic cells and enhance natural killer cell-mediated cytotoxicity (Maniar et al., 2010; McCarthy et al., 2013). 3) Promote target cell apoptosis: destroy the cell structure of target cells by secreting perforin and granzyme B; exert antibody-dependent cell-mediated cytotoxicity through certain membrane surface receptors such as FcγR; through Fas/FasL Pathways, expression-related apoptosis-inducing ligand CD95 ligand and TNF-related apoptosis-inducing ligand (TRAIL), etc. cause programmed apoptosis of target cells (Pennington et al., 2005; Poonia and Pauza, 2012). 4) Antigen presentation: partially activated γδT cells are specialized antigen presenting cells, and their surface highly expresses chemokine receptors CCR7, MHC-Ⅱ molecules, CD80 and CD86, etc., and processes the antigens and cross-presents them to αβT cells thus stimulate a specific immune response (Brandes et al., 2009; Wu et al., 2009). 5) Immune surveillance and immunomodulation: Activated γδ T cells exert immune surveillance through the high expression of CCR7 and CD161 on their surface (Chodaczek et al., 2012); through the production of IL-10, transforming growth factor-β (TGF-β) and other cells factors play an immunomodulatory role (Kühl et al., 2009). 6) Tumor-promoting effect (Figure 1): The tumor-promoting effect of γδT cells is mainly related to the production of IL-17. It not only induces tumor angiogenesis, stimulates tumor cell proliferation, and promotes tumor cell metastasis (Welte and Zhang, 2015; Qian et al., 2017), but also mobilizes pro-inflammatory neutrophils or immunosuppressive myeloid cells. Myeloid-derived suppressor cells inhibit the activation of CD8+T cells through high expression of ARG1 (Romano et al., 2018; Sacchi et al., 2018), and reactive oxygen species (ROS) produced by neutrophils have a certain inhibitory effect on IL-17-producing γδT cells (Wakita et al., 2010; Benevides et al., 2015; Coffelt et al., 2015). Other tumor-promoting effects of γδT cells include inhibiting the maturation of DCs, the senescent DCs can further suppress CD4+T cells and CD8+T cells (Peng et al., 2007; Ye et al., 2013a; Ye et al., 2013b), inhibiting T cell responses by secreting galectin and expressing programmed cell death protein ligand 1 (PDL1); and inducing tumor cell proliferation by expressing IL-22 and biregulin (Daley et al., 2016; Khosravi et al., 2018; Chen et al., 2019; Silva-Santos et al., 2019; Zhang et al., 2020). 7) Anti-tumor effect (Figure 2): Direct anti-tumor effect: activated γδΤ cells can secrete perforin, granzyme B and IFN-γ or express CD95 ligand (CD95L) and TRAIL to directly kill tumor cells (Gao et al., 2003); Indirect anti-tumor effect: Activated γδT cells induce DC maturation and infiltration by the secretion of TNF-α and IFN-γ (Conti et al., 2005; Münz et al., 2005; Nussbaumer et al., 2011); induce robust NK cell-mediated anti-tumor cytotoxicity through CD137 engagement (Maniar et al., 2010); efficiently processed and displayed antigens and provided co-stimulatory signals sufficient for strong induction of naïve αβT cell proliferation and differentiation (Brandes et al., 2005; Khan et al., 2014; Mao et al., 2014); γδT cells can target tumor associated macrophages and MDSCs to improve their anti-tumor ability. The enhanced killing capacity was correlated with the increased CD25 expression and IFN-γ secretion of γδT cells (Li et al., 2022).

## 3 γδT cells and tumors

### 3.1 Digestive system

1) Gastric cancer: Compared with normal gastric mucosa, the γδT cells in gastric cancer tissue are mainly Vδ1+T cells. The frequency of Vδ1γδT cells in gastric cancer tissue is reduced, the function is impaired, the secretion of IFN-γ and the expression of NKG2D are reduced. Reduced NKG2D expression may be one of the mechanisms of impaired function. The γδT cells infiltrated in gastric cancer tissue are also related to the prognosis of patients and can be used as an independent factor to judge the prognosis of patients (Kuroda et al., 2012; Wang. J et al., 2017; Chen et al., 2017). *In vitro*, γδT cells induced by pyrophosphate combined with IL-2 exhibited killing activity against gastric cancer cell line SGC-7901. The cytotoxicity of the cell line SGC-7901 was enhanced (Wu et al., 2016). Peripheral-derived γδT cells activated by gastric tumors can not only effectively kill tumor cells, but also induce the activation and proliferation of CD4^+^ and CD8+αβT cells through the antigen-presenting cell properties of Vγ9Vδ2 T cells, and enhance the cytotoxic function of CD8+αβT cells (Mao et al., 2014). *In vivo*, γδT cells also showed a certain anti-tumor effect, Wada et al. (Wada et al., 2014) used Vδ2+γδT cells induced and cultured *in vitro* to immunotherapy patients with advanced gastric cancer with malignant ascites. After treatment, the number of tumor cells in the patients’ ascites was significantly reduced and the ascites of some patients were controlled. Postoperative recurrence of gastric cancer is still a common problem, and cellular immunotherapy combined with chemotherapy seems to benefit patients after gastric cancer surgery. Oxaliplatin, a platinum drug for the treatment of gastric cancer, can upregulate the expression of NKG2D in tumor cells, thereby enhancing the sensitivity of tumor cells to kill mediated by γδT cells, NK cells, or cytokine-induced killer (CIK) cells (Gasser et al., 2005). When γδT cells, NK cells, and CIK cells combined with chemotherapy (5-FU and platinum) were used as adjuvant therapy after gastric cancer surgery, the adjuvant therapy showed good resistance compared with chemotherapy alone. Receptivity and safety can improve the quality of life of patients, significantly reduce the risk of recurrence and metastasis of stage II/III gastric cancer, and significantly improve the clinical prognosis of patients with stage II/III gastric cancer (Cui et al., 2015; Wang. Y et al., 2017).
2) Hepatocellular carcinoma: γδT cells can effectively kill a variety of liver cancer cell lines *in vitro* (Bouet-Toussaint et al., 2008; Hoh et al., 2013; Honda et al., 2015), and the presence of low concentrations of zoledronate can enhance the sensitivity of HCC cells to Vγ9Vδ2 T cell-mediated killing (Sugai et al., 2016). However, liver cancer infiltrating γδT cells have defects in killing function and secretion of IFN-γ. This defect is caused by the following factors such as the damage of “T cell receptor pathway”, “natural killer cell pathway” and “primary immunodeficiency pathway”. It may be caused by a large number of infiltrating Treg cells in liver cancer tissues. Treg cells directly inhibit the effector function of γδT cells through cytokines TGFβ and IL-10 (Yi et al., 2013). Studies have found that allogeneic Vδ2 + γδT cells can complement the loss of anti-tumor function of liver cancer-infiltrating γδT cells (He et al., 2022). HCC tissue-resident γδT cells exhibit the characteristics of tissue-resident memory T cells and can effectively target ZOL-sensitized HCC tumor cells (Zakeri et al., 2022). Higher frequency of intratumoral γδT cells in HCC is associated with improved survival in HCC patients (Zakeri et al., 2022), The presence of γδT cells in adjacent tissues is related to the recurrence rate of hepatocellular carcinoma patients after surgery. The ratio of peritumoral hepatic stellate cells (HSCs) to γδT cells affects the invasiveness and recurrence of hepatocellular carcinoma, and the levels of IFN-γ, IL-17 and TNF-α secreted by γδT cells increased after cultured in HSC-containing medium, and greatly reduced the proliferation and invasiveness of liver cancer cells (Cai et al., 2014; Zhou et al., 2019). Increasing the number and function of γδT cells may become a new way to treat hepatocellular carcinoma.
3) Colorectal cancer: The study of Wu et al. (Wu et al., 2014a) proved that Vδ1 T cells induced and cultured *in vitro* by PHA combined with IL-7 showed a significant inhibitory effect on NOD/SCID mouse transplanted tumors established with the human colon cancer cell line HT29. Vγ9Vδ2T cells isolated from ascites have a killing effect on most colon cancer cell lines. This effect is related to the accumulation of IPP in tumor cells and the expression of ICAM-1, but there is no effect on normal colon cells (Corvaisier et al., 2005). Most colorectal cancer tissues express chemokines CCR5 and CXCR3 ligands, and CCR5 and CXCR3 receptors are expressed on the surface of γδT cells. The combination of the two promotes the migration of γδT cells to the tumor tissue and migrates to the γδT cells around the tumor tissue. The binding of NKG2D receptor to MICA/B and ICAM-1 on the surface of colon cancer cells is activated. The activated γδT cells release perforin and granzyme B to secrete cytokines IFN-γ, TNF-α, as well as TRAIL, Fas/FasL and many other ways to exert an effect on colon cancer cells (Todaro et al., 2009; Wu et al., 2014a). However, B7-H3+γδT cells and γδT17 are present in colon cancer that play the opposite role. The proportion of γδT cells expressing the immunomodulatory protein B7-H3 (CD276) in the peripheral blood and tumor tissues of colon cancer patients was significantly increased, and B7-H3 inhibited T-bet (a transcription factor of the T-box gene family) in Vδ2 T cells by inhibiting to inhibit the expression of IFN-γ and Vδ2 T cytotoxicity by downregulating the expression of perforin/granzyme B (Lu et al., 2020). Tumor-infiltrating γδT17 cells are the main IL-17-producing cells in human colorectal cancer, and activated γδT17 cells promote the proliferation of PMN-MDSCs by secreting cytokines such as IL-17, IL-8, TNF-α and GM-CSF, and maintain its immunosuppressive activity, promoting tumor progression (Wu et al., 2014b). The study of γδT cells with different functions provides more possible potential immunotherapies for colon cancer.
4) Pancreatic cancer: Oberg et al. (Oberg et al., 2014) used pancreatic cancer cell lines PancTu-I and SCID-Beige mice to establish a nude mouse tumor model, and used γδT cells, IL-2 and [(Her2)2xVγ9] to treat the above-mentioned tumor-bearing mice. This study found that the tumors in all mice were inhibited, and the significantly inhibited, moderately inhibited, and slightly inhibited patients accounted for 2/5, 1/5, and 2/5, respectively. However, the γδT cells infiltrated in pancreatic ductal adenocarcinoma (PDA) have a promoting effect on the occurrence and development of tumors. The cells are widely distributed in the interstitium of PDA, accounting for about 75% of infiltrating T cells, and the effective memory γδT cells are the main ones. These cells highly express IL-10, IL-17, FoxP3, PDL1 and galectin 9 (Gal-9). The interaction of these factors can cause adaptive immune suppression, thereby promoting the occurrence and development of PDA (Daley et al., 2016).
5) Esophageal cancer: The killing effect of γδT cells on esophageal tumor cells is mainly related to the expression of HSP on the surface of tumor cells. There may be γδT cell subsets expressing two phenotypes (Vγ9/Vδ2, Vγ9/Vδ1) in the peripheral blood of patients with esophageal cancer. These γδT cells recognize the HSP-60 and HSP-70 that expressed on the surface of tumor cells, shows cytotoxicity against autologous and allogeneic esophageal cancer cells (Thomas et al., 2000). In two phase 1 clinical trials of adoptive cellular immunotherapy using autologous γδT cells for recurrent or metastatic esophageal cancer (r/mEC), γδT cells with or without chemotherapy (docetaxel, cisplatin, and 5-fluorouracil) (DCF) in combination with chemotherapy) are safe and feasible, and γδT cells combined with chemotherapy can benefit patient survival (Sato et al., 2021).

### 3.2 Reproductive system

1) Ovarian cancer: A number of studies have shown that, γδT cells have a killing effect on ovarian cancer cells. *In vitro*, polyclonal γδT cells proliferated and activated by *γ*-irradiated k562-derived artificial antigen-presenting cells (aAPCs) showed a wide range of anti-tumor activities, and had certain anti-tumor activity against various ovarian cancer cell lines, such as CAOV3, EFO21, UPN251, IGROV1, and OC314. There are obvious inhibitory effects on the transplanted tumor of NSG mice established with ovarian cancer cells CAOV3-effLuc-mKate, and significantly reducing the tumor burden in mice (Deniger et al., 2014). *In vitro*, Free or liposomal aminobisphosphonic acid salts such as zoledronic acid (ZA) and alendronic acid (AA) can enhance the killing effect of γδT cells on ovarian cancer cell lines SKOV-3 and IGROV1, and patient-derived γδT cells can also kill autologous cells after activation ovarian cancer cells. Intraperitoneal administration of low doses of AA or liposomal AA (L-AA) with γδT cells resulted in modest tumor regression in many SKOV-3-luc xenograft mice, and higher doses of AA or L-AA Intravenous administration with γδT cells resulted in marked and sustained tumor regression in SKOV-3-luc xenograft mice and prolonged survival of the mice. Activation of γδT cells by L-AA was also demonstrated in mice with a more aggressive IGROV-1-luc tumor model. The low maximum tolerated dose of liposomal ZA in SCID mice limits its application *in vivo* (Parente-Pereira et al., 2014). Foord et al. found that ascites-derived γδT cells had higher killing ability than CD8 + T cells in killing mature ovarian cancer cell line OVCAR-3, produced a higher proportion of IFN-γ, and derived from long-term survivors. γδT cells showed higher killing capacity than deceased patients (Foord et al., 2021). Another study found (Lai et al., 2012) that γδT cells also had an inhibitory effect on cells expressing stem cell markers in ovarian cancer. Researchers co-cultured γδT cells with microspheres with stem cell characteristics induced under certain conditions and found that the microspheres proliferation rate and the expression of stem cell-related genes were significantly reduced, the sensitivity to paclitaxel and cisplatin was increased, and the expression of antigens HLA-DR, B7-1, and B7-2 were significantly increased. It can be seen that γδT cells have a clear inhibitory effect on putative cancer stem cells.
2) Cervical cancer: One of the main risk factors for cervical cancer is the persistent infection of high-risk HPV. HPV-positive cervical cancer cells have low expression of MHC class I antigens, which limits the tumor recognition and anti-tumor effects of conventional T cells. The non-MHC restricted properties of γδT cells may play an important role in the immunotherapy of cervical cancer. In the research on cervical cancer, it was found that both bisphosphonate chemotherapeutics and galectin-1 (Gal-1) monoclonal antibody can enhance the anti-tumor effect of γδT cells. The immunosuppressive factor Gal-1 has been widely concerned. There is an inhibitory effect on the activity of γδT cells. When Gal-1 monoclonal antibody and γδT cells are used in combination with SiHa and HeLa cells *in vitro*, the killing activity of γδT cells is enhanced. Bisphosphonate chemotherapeutics such as pamidronate can increase the sensitivity of various cervical cancer cell lines such as HeLa, SiHa and CaSki to Vγ9Vδ2T cells, and enhance the antitumor activity of γδT cells. The enhancing effect of Gal-1 monoclonal antibody and bisphosphonate chemotherapeutics on the anti-tumor activity of γδT cells has been confirmed in mouse tumor-bearing experiments (Li et al., 2010; Lertworapreecha et al., 2013). However, it is worth noting that there are γδT17 cells in HPV-related cervical squamous cell carcinoma, which play a role in promoting the occurrence and development of tumors (Van Hede et al., 2017).
3) Prostate cancer: The use of syngeneic γδT cells for adoptive immunotherapy in a mouse prostate cancer model inhibited the growth of cancer cells (Liu et al., 2008). Zol combined with IL-2 *in vivo* expansion γδT cell therapy has a certain effect on hormone refractory prostate cancer. The effect is related to the maintenance or increase of the number of γδT cells during the treatment period. Moreover the increased γδT cells were mainly CD45RA-CD27-effect memory type and CD45RA+CD27-terminally differentiated type with direct effector functions and cytotoxic effects (Dieli et al., 2007). The inhibitory effect of γδT cells on prostate cancer has been confirmed in mice and clinical trials. However, the inhibitory effect of γδT cells on prostate cancer is different among different prostate cancer cell lines. DU145 is sensitive to the cytotoxicity of γδT cells, while PC-3 is characterized by its low activity of the melanic acid pathway and low IPP content in the body without sensitive (Arkko et al., 2015). Therefore, when using bisphosphonates to activate γδT for tumor immunotherapy, the type of tumor cells is an important consideration.

### 3.3 Urinary system

The research on the effect of γδT cells on urinary system tumors is mainly in kidney cancer. Peripheral blood γδT cells of patients with metastatic renal cell carcinoma (MRCC) can selectively act on kidney cancer cells after being activated by nitrogen-containing bisphosphonate and IL-2, but there are no effect on normal kidney cells. The above-mentioned selectivity may be related to the high expression of MICA/B and ULBP in renal cancer cells. γδT cells combine with MICA/B and ULBP through the NKG2D receptor to provide costimulatory signals to enhance the lysis of tumor cells by TCR signals (Viey et al., 2005). Zoledronate combined with IL-15 to induce γδT cells from healthy volunteer PBMCs effectively inhibited the growth of tumors in mice bearing renal cell carcinoma patient-derived xenografts and prolonged the survival time of tumor-bearing mice (Zhang et al., 2021).

The inhibitory effect of γδT cells on renal cell carcinoma has also been studied in a small clinical scale. Kobayashi et al. (Kobayashi et al., 2007) used γδT cells to treat patients with advanced renal cancer after radical nephrectomy. Among the 7 patients, 3 patients had prolonged tumor doubling time and increased the number of γδT cells in peripheral blood. The study also found that the proportion of γδT cells in the peripheral blood of patients was related to the rate of tumor metastasis and the occurrence of tumor-related deaths in patients, which is one of the important factors to improve the prognosis of renal cancer patients. Bennouna et al. (Bennouna et al., 2008) used γδT cells combined with IL-2 to immunotherapy in 10 patients with metastatic renal cell carcinoma. 6 patients were in stable condition and the tumor progression time was prolonged. The immunotherapy of γδT cells may be the gospel for patients with advanced renal cancer.

### 3.4 Respiratory system

1) Nasopharyngeal carcinoma: Zheng B et al. (Zheng et al., 2001a) experimentally confirmed that γδT cells obtained by selective expansion of healthy human peripheral blood mononuclear cells *in vitro* had a certain cytotoxic effect on nasopharyngeal carcinoma cell lines CNE2 and 915, and this effect was related to the number of CD56-γδ T cells. Zheng BJ et al. (Zheng et al., 2001b) found that the use of nasopharyngeal carcinoma cell line CNE2 to establish a nude mouse tumor model, 5 days after CNE2 cell inoculation, tumors were seen subcutaneously in nude mice, and nude mice that were not treated with γδT cells had progressive tumors growth, the average lifespan of mice was 35 ± 3.4 days; CNE2 cells were inoculated with γδT cell treatment on the 10th day, and only a single dose of γδT cell treatment was given to the group. The tumor resumed growth 1 week later, and the average lifespan of mice was 61 ± 15.7 days; Once every other week, the group who was given γδT cell treatment twice resulted in delayed tumor recovery and growth, and the average life span of mice was prolonged by 74 ± 12.9 days. The results of immunohistochemistry showed that the tumor specimens on the second day of γδT cell treatment showed γδT cell accumulation and local necrosis, while on the sixth day, the infiltrating cells in the tumor tissue disappeared and the tumor cell mitosis increased. The above studies suggest that γδT cells exert a certain inhibitory effect on nasopharyngeal carcinoma cell lines both *in vivo* and *in vitro*.
2) Lung cancer: The inhibitory effect of γδT cells on lung cancer has been confirmed *in vitro*, mice and lung cancer patients. *In vitro* studies have shown (Xie et al., 2018) that the expanded γδT cells have a certain killing effect on the lung squamous cell line SK-MES-1 and the lung adenocarcinoma cell line A549; the use of *in vitro* expanded γδT cells on the human lung cancer cell A549, the mice were subjected to immunotherapy and found that the tumor growth rate slowed down, and the tested mice had no acute adverse reactions; Nakajima et al. (Nakajima et al., 2010) used γδT cells to perform adoptive immunotherapy on 10 patients with advanced lung cancer, and 3 patients were in stable condition. The inhibitory effect of γδT cells on lung cancer was related to the expression of HSP72 on the surface of lung cancer cells to varying degrees, a platelet-like receptor with a relative molecular mass of 67000 and a high affinity, and human MutS homologous protein 2 (hMSH2) molecules. After the T cell receptor TCRγδ or the natural killer receptor NKG2D is recognized, γδT cells are activated, and the levels of the activated γδT cells expressing CD69 and CD107a are significantly increased, and the secretion of IFN-γ and TNF-α increases, thereby killing and eliminating target cells (Ferrarini et al., 1996; Wei et al., 2018). The effects of γδT cells on lung cancer cells have been studied in depth, and how to better apply them in clinical practice needs to be further studied.
3) Breast cancer: The most effective treatment for breast cancer is surgery. Chemotherapy and immunotherapy are indispensable consolidation treatments after surgery. Studies have found that γδT cells have different inhibitory effects on different breast cancer cell lines. They have obvious inhibitory effects on breast cancer cell lines SkBr7, MCF7 and MDA-MB-231, while their inhibitory effects on BrCa-MZ01 are not obvious. The existence of this phenomenon may be related to the expression level of MICA/B and ICAM1 on the surface of breast cancer cells. γδT cells can up-regulate the expression of MICA/B and ICAM1 on the surface of the sensitive strain SkBr7. These molecules bind to the NKG2D receptor on the surface of γδT cells to trigger changes in intracellular signal molecules, protein kinases AKT, ERK and other signal molecules related to cell proliferation. The phosphorylation level of signal transduction and activator of transcription 3 (STAT3) decreased, and the expression level of pro-apoptotic molecules such as PARP and Caspase3 increased. Therefore, the recognition and binding of NKG2D receptors of γδT cells with MICA molecules expressed by tumor cells may be a necessary condition for their anti-tumor effects. The immunotherapy of NOD/SCID mouse xenograft model established by the sensitive strain SkBr7 and the resistant strain BrCa-MZ01 using γδT cells showed that γδT cells had a significant inhibitory effect on the tumor formation of the sensitive strain SkBr7, which was manifested by accelerated tumor cell apoptosis. Angiogenesis was inhibited, and tumor burden decreased. The non-sensitive cells did not appear to be suppressed. The appearance of these phenomena was not only related to the above analysis factors, but also related to the secretion of chemokines, tumor macrophage infiltration, etc. They act together on the tumor microenvironment, enhance the immune surveillance of tumors, inhibit tumor cell proliferation and induce them apoptosis (Aggarwal et al., 2013). In addition, activated γδT cells secreted IFN-γ, stimulated cancer stem cells (CSCs) to up-regulate the expression of MHC class I molecules and ICAM-1, and enhanced the killing effect of CD8+T cells, both of which synergistically targeted breast cancer stem-like cells (Chen et al., 2017). The study of these mechanisms provides a theoretical basis for the clinical application of γδT cells. Meraviglia et al. (Meraviglia et al., 2010) used zoledronic acid combined with IL-2 *in vivo* proliferation and activation of γδT cells for immunotherapy of 10 patients with advanced breast cancer, and found that the progression of the patient’s condition was related to the number of peripheral Vγ9Vδ2 T cells, and the condition was partially relieved or stable. The number of Vγ9Vδ2 T cells in the peripheral blood of 3 patients was maintained at a high level and the level of CA153 decreased. The number of Vγ9Vδ2 T cells in the peripheral blood of the 7 patients whose condition deteriorated could not be maintained continuously. Another study found that γδT cells could enhance the efficacy of trastuzumab in patients with HER-2 positive breast cancer, and the tumor volume of patients was significantly reduced (Capietto et al., 2011). Rukangyin and its disassembled prescriptions can inhibit the proliferation of triple-negative breast MDA-MB-231 cells and induce their apoptosis (Li et al., 2020). After Rukangyin activates γδT, it improves the killing rate of breast cancer MDA-MB-231 cells (Li et al., 2020; Chen et al., 2021). It can be seen that immunotherapy based on γδT cells may become a new method for breast cancer treatment.

### 3.5 Nervous system

The research on the effect of γδT cells on nervous system tumors is more common in neuroblastoma (neurobiastoma, NB). Many studies have confirmed (Schilbach et al., 2000; Chargui et al., 2010) that γδT cells proliferated and activated *in vitro* are highly cytotoxic to human neuroblastoma cells. They can effectively kill a variety of NB cell lines. γδT cells mainly recognize the target of NB cell line through their TCRγδ, and NKG2D has a weak role in NB cell lysis. This may be related to the lack of MICA on NB cells. These studies show the feasibility of using γδT cells to treat patients with neuroblastoma. Pressey et al. (Pressey et al., 2016) used phosphoantigen combined with IL-2 to perform immunotherapy on patients with refractory neuroblastoma and found that the therapy was well tolerated. The number of γδT cells in the patient increased significantly, and the patient had experienced remission. No toxic side effects of the therapy were found.

### 3.6 Blood system

γδT cells not only show inhibitory effects on solid tumors, but also have clear killing activity on hematological tumors. γδT cells are effective against many types of acute leukemia cell lines such as Jurkat cell line, THP-1 cell line, HL-60 cell line, chronic myeloid leukemia K562 cell line, multiple myeloma RPMI-8226 cell line and histiocytic lymphoma U-937 cell line, and it has strong killing effect on Jurkat cell line and U-937 cell line (Xiao et al., 2017). D'Asaro et al. (D’Asaro et al., 2010) confirmed that Vγ9Vδ2 T cells could recognize, phagocytose and effectively kill imatinib-sensitive CML cell line K562S and imatinib-resistant CML cell line K562R after pretreatment with zoledronate, KCL22R and LAMA84R, and prolong the survival of Nod/SCID mice bearing the CML cell line MM-1. Almeida et al. (Almeida et al., 2016) found that DOT cells had high cytotoxicity to CLL cell line MEC-1, autologous and allogeneic CLL cells, and had a significant inhibitory effect on NSG mouse xenografts established by MEC-1 cell line. DOT cells selectively target transformed B lymphocytes through their specific TCR mechanism and NKR mechanism, but there was no effect on normal B lymphocytes. DOT cells also showed a certain inhibitory effect on various AML cell lines such as THP-1, HEL, AML-193, MV4-11, HL-60, U-937, OCI-AML3, Kasumi-1 and KG-1, did not respond to normal leukocytes including CD33^+^ or CD123+ myeloid cells. Adoptive cell therapy with DOT cells reduces AML burden in blood and target organs in various human AML xenograft models and significantly prolongs host survival without significant toxic effects. DOT cells can also target chemoresistance AML cells. These provide a theoretical basis for the application of DOT cells in the treatment of CLL and AML (Lorenzo et al., 2019). γδT cells can directly kill leukemia cells through perforin/granzyme-dependent cytolysis, and they can also act on hematological tumors by secreting cytokines IFN-γ and TNF-α (Gertner-Dardenne et al., 2012; Xiao et al., 2017). Tokuyama et al. (Tokuyama et al., 2008) found that the killing effect of γδT cells on lymphoma was related to the expression level of CD16 molecules on the cell surface. Trastuzumab and tuximab can enhance the killing activity of γδT cells against lymphoma. Wilhelm et al. (Wilhelm et al., 2003) used pamidronate (PAM) combined with IL-2 proliferation and activation of γδT cells in patients with immunotherapy of 19 patients with refractory non-Hodgkin’s lymphoma and multiple myeloma, and found that PAM combined with low. The dose of IL-2 can specifically induce the proliferation of γδT cells, so that the patient’s condition was stable or partially relieved without obvious adverse reactions. These studies have laid the foundation for the clinical application of γδT cells for immunotherapy of hematological tumors.

### 3.7 Other

Jiang Hui et al. (Jiang et al., 2010) found that γδT cells had a strong killing effect on the osteosarcoma cell line HOS, whether *in vitro* or in tumor-bearing mice. Studies by Lozupone et al. (Lozupone et al., 2004) confirmed that both *in vivo* activation and adoptive infusion of γδT cells could inhibit the growth of tumors in melanoma-bearing mice and prolong the survival time of tumor-bearing mice. Malignant melanoma cell lines express NKG2D ligand and low expression of MHC-I related antigen A. These molecules bind to the NKG2D receptor of γδT cells to activate the body’s anti-tumor immunity. Compared with healthy people, the peripheral blood γδT cells of melanoma patients increased significantly, and the increased γδT cells were mainly CD3 + CD28-γδT cells, which exerted anti-tumor effects through the expression of a large amount of perforin (Campillo et al., 2007). In B16 melanoma, IFN-γ produced by γδT cells serves as an early and important source of IFN-γ in tumor immune surveillance, plays a critical role in protecting immune responses against tumor development, and modulates tumor antigen-triggered CD4^+^ and CD8^+^ T cells response, thereby enhancing the recognition and potency of cytotoxic T cells against cancer cells (Khan et al., 2014).

## 4 γδT-cell-based cellular strategies

Evaluation of adoptive transfer and *in vivo* amplification of Vδ2+T cell efficacy Phase II trials showed that although Vδ2 + T Cells continue to activate and proliferate, but the clinical response of solid tumors is limited (Dieli et al., 2007; Bennouna et al., 2008; Kobayashi et al., 2011; Lang et al., 2011; Noguchi et al., 2011; Sakamoto et al., 2011; Scheper et al., 2014; Lo Presti et al., 2017; Zou et al., 2017; Hoeres et al., 2018). The mechanisms of preventing activation of Vδ2 + T cells inducing long-term anti-tumor immunity in cancer (Hayday and Tigelaar, 2003; Pennington et al., 2006; Kabelitz et al., 2013; Wesch et al., 2014; Peters et al., 2018) include the immunosuppressive function of γδT cells (Wu et al., 2014b; Daley et al., 2016), especially after TCR stimulation in different environments (Casetti et al., 2009; Traxlmayr et al., 2010; Peters et al., 2014), a study showed that TCR stimulation alone led to immunosuppressive behavior, and the degree of immunosuppression was related to the intensity of TCR signal (Schilbach et al., 2020). Even a single TCR crosslinking will produce inhibition behavior (Schilbach et al., 2020). However, several new immunotherapy strategies based on γδT cells have emerged. The application of γδT cells in tumor immunotherapy brings new hope. Almeida et al. (Almeida et al., 2016) used a 3-week culture program to obtain DOT cell products that showed inhibitory effects on a variety of CLL and AML cell lines; Chimeric antigen receptor (CAR) γδT cells can improve the efficacy of CAR-T cells and reduce their side effects (Rischer et al., 2004; Harrer et al., 2017; Ang et al., 2020; Rozenbaum et al., 2020; Makkouk et al., 2021; Sánchez Martínez et al., 2022); Wallet et al. described the generation of induced pluripotent stem cell-derived γδ CAR-T-cells (γδCAR-iT). They demonstrated a single dose of γδ CAR-T-cells resulted in potent tumor growth inhibition in a xenograft mouse model (Wallet et al., 2021); Bispecific γδT lymphocyte conjugator (bsTCE) optimizes Vγ9Vδ2 Tumor targeted activation of T cells not only preserves the ability of immune cells to recognize and kill tumors, but also promotes the immune response against tumors (Labrijn et al., 2019); Humanized anti BTN3A (also called CD277) monoclonal antibody can selectively activate Vγ9Vδ2 T cells, and further stimulate the immune system to kill tumor cells (Harly et al., 2012). These therapeutic strategies show promising anti-tumor activity *in vitro* and *in vivo* (Table 1). These therapeutic strategies will be evaluated in Phase I/Phase II clinical trials (Table 2), and the results of these trials will determine whether the potential of γδT cells can be translated into clinical benefits.

**TABLE 1:** Preclinical trials based on γδT-cells.

Year	Author	Effector cells	Tumor type	Outcome
2016	Almeida Almeida et al., 2016	DOT (Vδ1T)	CLL-MEC-1 NSG mice, various human AML xenograft models	prolongs host survival without significant toxic effects
2022	Sánchez Martínez Sánchez Martínez et al., 2022	CD123 CAR-DOT (Vδ1T)	AML-PDX^Luc^ NSG mice	complete control of leukemia in mice
2004	Rischer Rischer et al., 2004	CD19 CAR-γδT (Vγ9Vδ2T) G_D2_ CAR-γδT (Vγ9Vδ2T)	Raji Reh LAN-1 JF	Efficiently and specifically lyse antigen-expressing tumour cells
2017	Harrer Harrer et al., 2017	gp100/HLA-A2 TCR-γδT MCSP CAR-γδT (Vγ9Vδ2T)	Mel 526 A375M	specifically lyse melanoma cells
2020	Ang Ang et al., 2020	NKG2D_Z_ CAR-γδT (Vγ9Vδ2T)	HCT116-Luc NSG mice SKOV3-Luc NSG mice	1/5 tumor growth inhibition,4/5 tumor growth slowing down; median survival extend significantly
2020	Rozenbaum Rozenbaum et al., 2020	CD19 CAR-γδT (Vγ9Vδ2T)	CD19^+/−^tumor cells B- ALL-Nalm6 NSG mice	highly reactive against good anti-leukemic activity but limited persistence of γδ CAR-T cells
2021	Makkouk Makkouk et al., 2021	GPC-3 CAR/SIL-15 Vδ1T	HCC-HepG2-NSG mice	controlled tumor growth
2021	Wallet Wallet et al., 2021	γδ CAR-iT	B-ALL-Nalm6 NSG mice	potent tumor growth inhibition

CAR, chimeric antigen receptor; G_D2,_ ganglioside antigen; LAN-1 JF, neuroblastoma cell lines; Raji, Burkitt’s lymphoma cell lines; Reh acute lymphocytic leukaemia cell lines; Mel526 A375M, melanoma cell lines; gp100, glycoprotein 100; MCSP, melanoma associated chondroitin sulfate proteoglycan; HCT116, Colorectal cancer cell lines; SKOV3, ovarian cancer cell lines; +, Positive; -, negtive; GPC-3, Glypican-3; HCC, hepatocellular carcinoma; γδ CAR-iT, Pluripotent stem cell-derived γδ CAR-T-cells.

**TABLE 2:** Ongoing clinical trials based on γδT-cells.

Title	Cellular strategies	Intervention	Malignancy	Phase	Organization	Start date	Recruitment status	Study identifier
A Safety and Efficacy Study of ADI-001, an Anti-CD20 Allogeneic Gamma Delta CAR-T, in Subjects With B cell Malignancies (GLEAN-1)	γδ CAR- T Cells	ADI-001. Anti- CD20 CAR-T + Lymphodeple-tion	B-NHL	I	Adicet Bio, Inc	4 March2021	Recruiting	NCT 04735471
Study of GDX012 in Patients With MRD Positive AML	Allogeneic γδT-cell transfer	GDX012. Allogeneic cell therapy enriched for Vd1+	AML	I	Gamma Delta Therapeutics Limited	13 August 2021	Teminated	NCT 05001451
Safety and Efficacy of *Ex-vivo* Expanded Allogeneic γδ T-lymphocytes (OmnImmune^®^) in Patients With Acute Myeloid Leukaemia (AML)	Allogeneic γδT-cell transfer	OmnImmune^®^	AML	I	TC Biopharm	Novem-ber 27, 2018	Completed	NCT 03790072
Trial of LAVA-051 in Patients With Relapsed/Refractory CLL, MM, AML	Antibody-based strategies	LAVA-051. Bispecifific γδT-cell engager	CLL, AML, MM	I/II	Lava Therapeutics	12 July 2021	Recruiting	NCT 04887259
Trial of LAVA-1207 in Patients With Therapy Refractory Metastatic Castration Resistant Prostate Cancer	Antibody-based strategies	LAVA-1207. Bispecifific γδT-cell engager	mCRPC	I/IIa	Lava Therapeutics	27 June 2022	Recruiting	NCT 05369000
Phase 1/2a Study of ICT01 Plus Low Dose SC IL-2 in Patients With Advanced Solid Tumors (EVICTION-2)	Antibody-based strategies	ICT01.anti-BTN3A mAb + IL-2	Solid Tumor, Adult	I/IIa	ImCheck Therapeutics	19 April 2022	Recruiting	NCT 05307874
A Study to Investigate the Safety and Effificacy of TEG002 in Relapsed/Refractory Multiple Myeloma Patients	Alternative γδT-cell-related strategies	TEG002	RR MM	I	Gadeta B.V.	13 May 2021	Active, not Recruiting	NCT 04688853

B-NHL, B cell Non-Hodgkin lymphoma; AML, acute myeloid leukemia; CLL, chronic lymphocytic leukemia; MM, multiple myeloma; mCRPC, metastatic castration resistant prostate cancer.

## 5 Conclusion

The role of γδT cells in tumor immunotherapy has gradually been recognized, but due to the lack of continuous and effective amplification methods, the complexity of γδT cell secretion factors, the various inhibitory factors existing in tumors, and the complexity of tumor microenvironment, etc. Existence limits the anti-tumor effect of γδT cells. How to establish and optimize a continuous and effective amplification method and further clarifying its mechanism of action is the direction of the researchers’ unremitting efforts. The synergistic anti-tumor effect between chemotherapeutic drugs and γδT cells provides new ideas for the application of γδT cells. In clinical applications, whether γδT cell immunotherapy, radiotherapy, surgery and other combined treatments are synergistic and whether they can improve the prognosis of patients is also one of the future research directions.
